# Protein Stacking on the APTES-Functionalized Pyrochlore Bi_2_Ru_2_O_7_ Clusters for Ultrasensitive and Selective Immunosensing

**DOI:** 10.1021/acsami.4c17869

**Published:** 2025-02-06

**Authors:** Nikola Tasić, Nika Vranešič, Dino Metarapi, Kristina Mervič, Milan Žunić, Aleksandra Dapčević, Matjaž Finšgar, Samo B. Hočevar

**Affiliations:** † Department of Analytical Chemistry, National Institute of Chemistry, Hajdrihova 19, 1000 Ljubljana, Slovenia; ‡ University of Belgrade, Institute for Multidisciplinary Research, Kneza Višeslava 1, 11030 Belgrade, Serbia; § Department of General and Inorganic Chemistry, Faculty of Technology and Metallurgy, University of Belgrade, Karnegijeva 4, 11000 Belgrade, Serbia; ∥ Faculty of Chemistry and Chemical Engineering, University of Maribor, Smetanova 17, 2000 Maribor, Slovenia

**Keywords:** Bi_2_Ru_2_O_7_ pyrochlore, APTES, SARS-CoV-2, spike protein, immunosensor, electrochemical, impedimetric

## Abstract

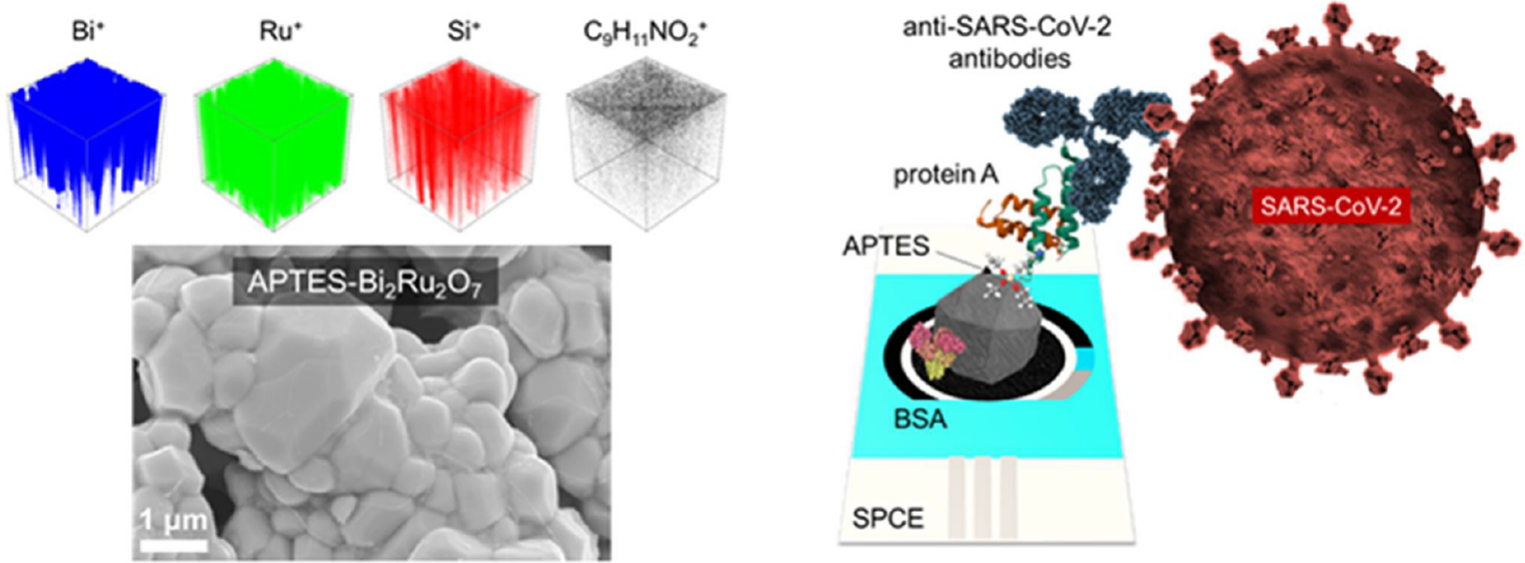

With their unique physicochemical properties, such as metallic-like conductivity, favorable (electro)catalytic properties, electrochemical stability, and ease of functionalization, pyrochlores have found applications in various fields such as solid oxide fuel cells, batteries, thick film resistors, and temperature sensors; however, there are no reports on their application in electrochemical immunosensing. In this study, we exploited the (electro)catalytic nature and stability of the pyrochlore Bi_2_Ru_2_O_7_ clusters silanized with (3-aminopropyl)triethoxysilane (APTES) to demonstrate their potential for the effective stacking of functional proteins. Characterization of the clusters by XPS disclosed a dual environment of Bi, also indicating the presence of Bi_2_O_3_ alongside APTES-Bi_2_Ru_2_O_7_ clusters and, importantly, the predominant involvement of pyrochlore moieties in subsequent protein stacking. After stacking protein A and antibodies, the immunosensor revealed a nearly interference-free operation, high sensitivity, a detection limit of 118 fM SARS-CoV-2 spike protein, and operation in a wide examined concentration range of 10^−5^−10^−1^ μg mL^−1^ with an *r*^2^ of 0.98. In combination with a short incubation time of 30 min, the pyrochlore-based immunosensor provides a solid platform for future point-of-need applications.

## Introduction

Since the early works,^1,2^ pyrochlore-type compounds of general formula A_2_Ru_2_O_7-δ_ (A = Pb, Bi) have attracted notable attention considering the energy-related and electronic applications, such as temperature sensors^3^ and thick film resistors.^4−6^ Of particular interest is bismuth-ruthenate (Bi_2_Ru_2_O_7_), which exhibits an effective bifunctional (electro)catalytic nature for both oxygen evolution reaction (OER) and oxygen reduction reaction (ORR), and it is characterized by a highly stable structure during electrocatalysis,^7^ thus making it a perfect candidate for sodium-air batteries.^8^ Its electrical and catalytic properties, similar to those of RuO_2_, have nominated it as a cathode material in solid oxide fuel cells.^9,10^ In addition, a recent report indicates its use in a nanocomposite with Pd particles and BiVO_4_ for the photocatalytic degradation of trichloroethylene.^11^ However, despite its apparent catalytic superiority and excellent stability, scrutinized in a recent study,^7^ the application of pyrochlores in sensor development is almost nonexistent. A solid electrolyte sensor using a sodium superionic conductor, i.e., NASICON, and a pyrochlore-type oxide (Pb_2_Ru_1.9_V_0.1_O_7−δ_) electrode was found promising for the potentiometric sensing of gaseous NO and NO_2_ at 400 °C,^12^ being the only thus far reported pyrochlore-based electrochemical sensor.

Electrochemical immunosensors have proven highly effective due to their enhanced sensitivity, selectivity, cost-effectiveness, quick response, and suitability for on-site applications.^13−15^ The SARS-CoV-2 virus has exemplified the critical importance and necessity of effective point-of-care immunosensors and assays for rapid and accurate detection in combating and controlling infectious disease outbreaks. Various types of (nano)materials have been explored for this purpose, including metallic nanoparticles,^16^ metal oxides,^17^ carbon-based nanomaterials,^18^ 2D-layered MXenes,^19^ nanocomposites,^20^ etc. Alternatively, SARS-CoV-2 can be detected via colorimetric assays,^21^ lateral flow assays,^22^ molecular detection methods such as PT−PCR,^23^ LAMP,^24^ or CRISPR-based detection.^25^

Here, we show for the first time how (electro)catalytic and silanized APTES-Bi_2_Ru_2_O_7_ clusters deposited on a screen-printed carbon electrode (SPCE) are ideally suited for further immobilization of the biological recognition element, i.e., the anti-SARS-CoV-2 antibodies, and enable highly sensitive impedimetric detection of the SARS-CoV-2 spike protein. The silanization with APTES was carried out to enable the binding of the pyrochlore clusters to the −OH groups of the HNO_3_-pretreated supporting electrode while enabling, on the other side, cross-links to protein A via glutaraldehyde (GA). GA is used as a cross-linking agent due to its high reactivity toward amino, hydroxyl, and carboxyl groups, whereas protein A provides a site-directed end-on fab-up immobilization of the anti-SARS-CoV-2 antibodies via their Fc fragments. In addition, the interference-free operation of the immunosensor was confirmed in the selectivity study with potential interferents such as the MERS, HCoV-OC43, HCoV-HKU1, HCoV-NL63, and HCoV-229E spike proteins.

## Results and Discussion

### Pyrochlore Characterization

**Figure 1:**
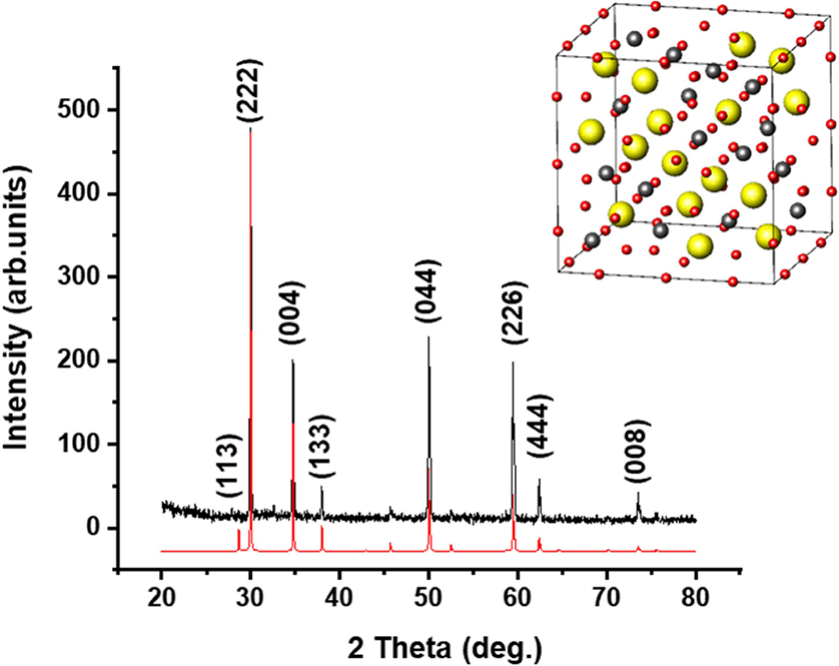
XRD pattern of the synthesized Bi_2_Ru_2_O_7_ powder. The black spectrum corresponds to synthesized Bi_2_Ru_2_O_7_ particles, while the red spectrum corresponds to the powder diffraction pattern for dibismuth(III) diruthenate(IV), classified under ICSD #78114.^26^ The inset shows the theoretical crystal structure of the cubic Bi_2_Ru_2_O_7_, with Bi atoms in yellow, Ru atoms in gray, and O atoms in red.

The powder XRD pattern (Figure 1) of a synthesized Bi_2_Ru_2_O_7_ sample can be indexed as cubic pyrochlore-type oxides (space group *Fd*3̅*m*). There are two crystallographic cation sites in the Bi_2_Ru_2_O_7_ crystal structure, i.e., 16d and 16c (inset of Figure 1); the Bi atom surrounded by eight O atoms in a distorted dodecahedron is located at 16d, while RuO_6_ octahedra occupy 16c. The observed diffraction peaks at 27.9, 30.0, 34.8, 38.0, 50.0, 59.5, 62.4, and 73.5° are assigned to (113), (222), (004), (133), (044), (226), (444), and (008) crystallographic planes, respectively.^8^ No prominent peaks in the powder XRD pattern corresponding to other phases or oxides were observed; this was also supported later by EDX analysis and the observed ratio between the metallic centers. The crystallite size of the synthesized material was 66.1 nm, calculated by Scherrer’s equation.

**Figure 2:**
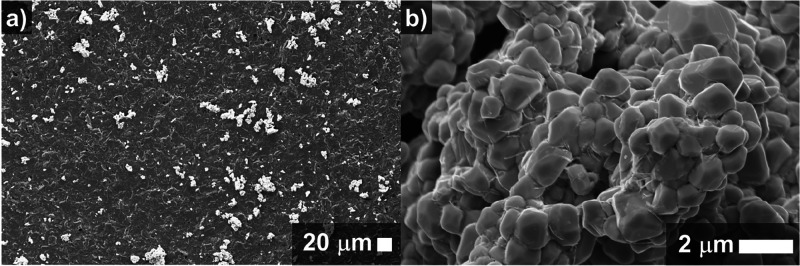
FE-SEM images of an SPCE modified with pyrochlore clusters. (a) APTES-Bi_2_Ru_2_O_7_ clusters on the SPCE (concentration of the drop-casting solution was 5 mg mL^−1^ in dH_2_O), and (b) APTES-Bi_2_Ru_2_O_7_ cluster at high magnification. The morphology analyses carried out on the bare SPCE are presented in Figure S1.

Insights into the structure of APTES-Bi_2_Ru_2_O_7_ clusters were obtained by field-emission scanning electron microscopy (FE-SEM) analysis, as shown in Figures 2 and S2. The submicrometer particles exhibit an irregular shape, resembling a rhombicuboctahedral structure, a polyhedron with triangular, square, and rectangular faces.

**Figure 3:**
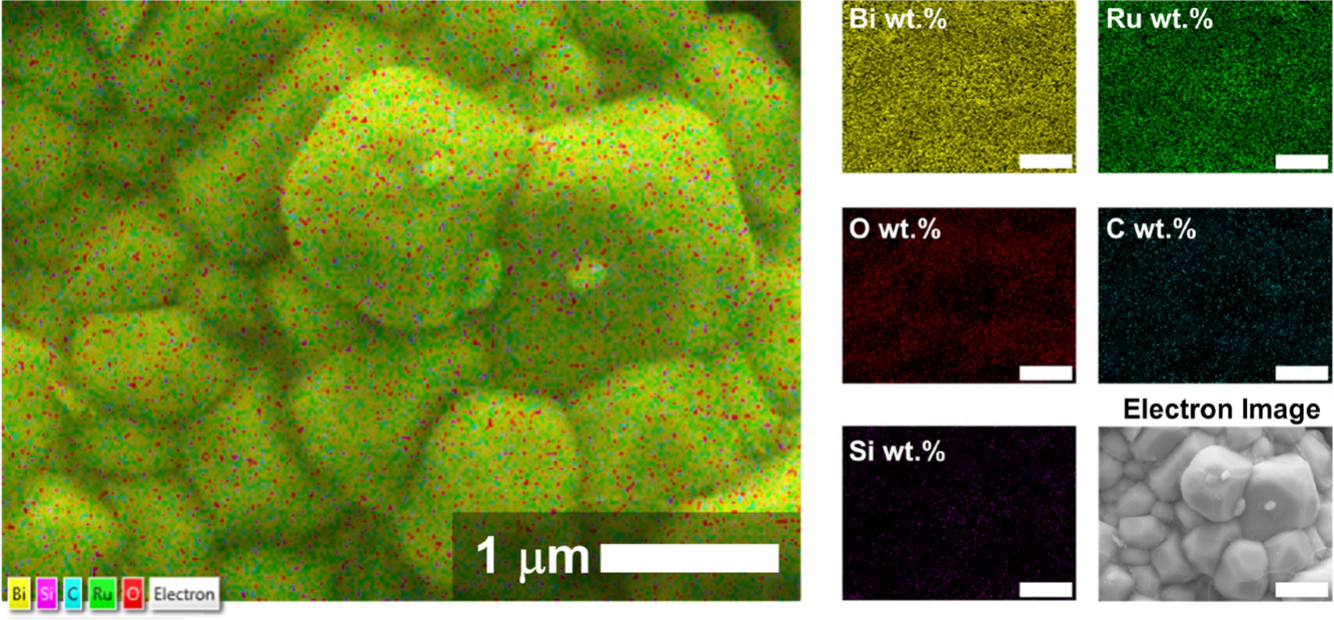
Elemental distribution in APTES-Bi_2_Ru_2_O_7_ clusters. The size bars on the right-side micrographs correspond to 1 μm.

We also performed EDX analysis on the APTES-Bi_2_Ru_2_O_7_ clusters, and the corresponding results are shown in Figure 3. Carbon and silicon are attributed to APTES as aminosilanes with a gross molecular formula of C_9_H_23_NO_3_Si. Highly magnified images of APTES-Bi_2_Ru_2_O_7_ clusters revealed superficial features resembling a spider web, likely originating from APTES (Figure S2).

The atomic ratio between Bi and Ru is stoichiometric with respect to the theoretical formula of Bi_2_Ru_2_O_7_, with an almost perfect match of the expected and measured atomic percentages, i.e., 18.2 vs 17.4 at. % for Bi and 18.2 vs 17.3 at. % for Ru, respectively. A slightly lower percentage of oxygen compared to the theoretical value is a common and highly desirable feature of pyrochlore structures, indicating the presence of oxygen vacancies.^27^ The corresponding atomic and weight distributions are shown as graphs in Figure S3.

### Electrochemical Studies of the Pyrochlore-Modified Electrode

**Figure 4:**
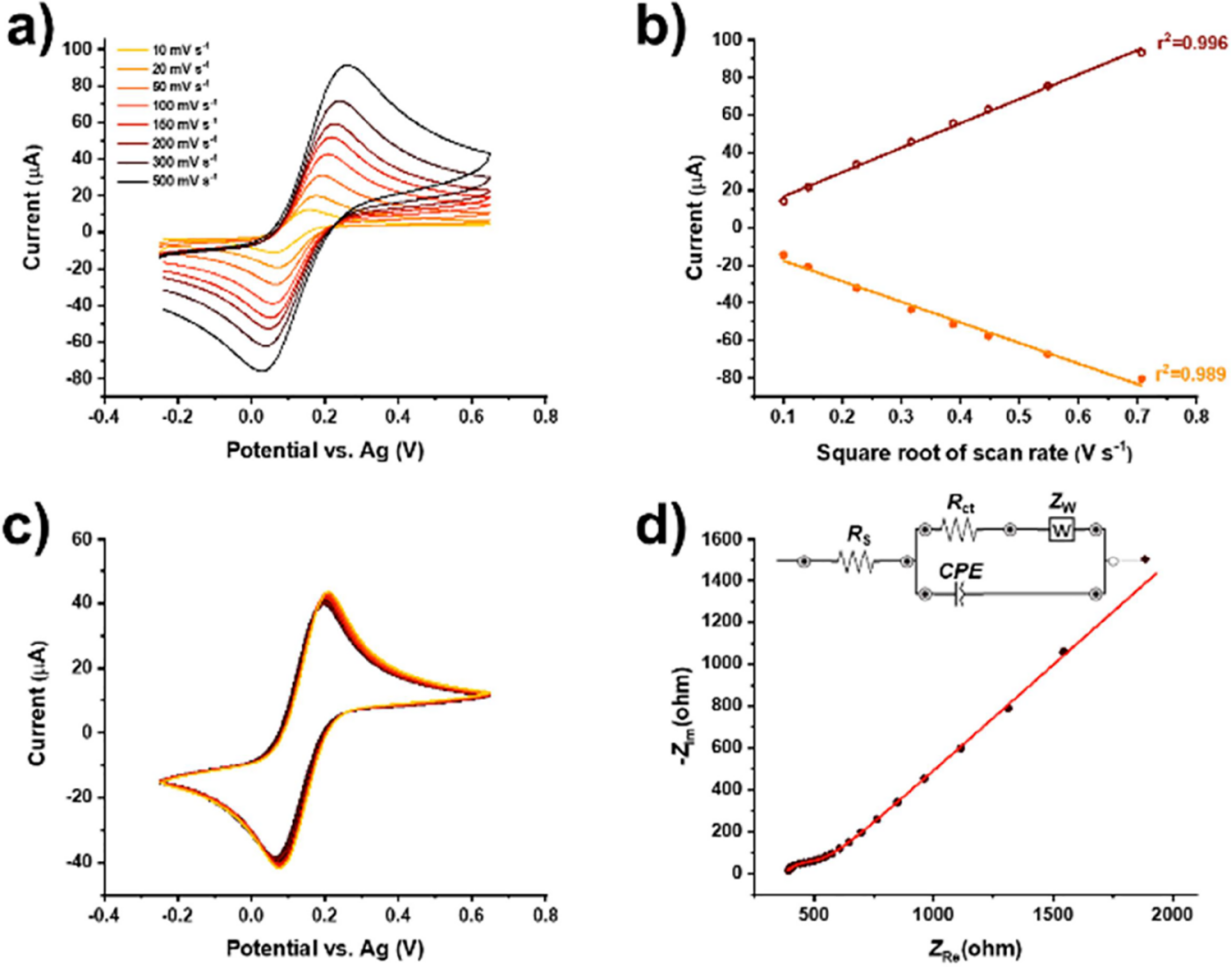
Electrochemical characterization of APTES-Bi_2_Ru_2_O_7_ cluster-modified SPCE in relevant physiological media. (a) CV measurements using different scan rates and (b) the corresponding oxidation and reduction peak currents with respect to the square root of the scan rate, (c) 30 consecutive CVs, and (d) EIS measurement in 1.0 mM [Fe(CN)_6_]^3−/4−^ in 0.1 M KCl (dots represent the measured and the solid line represents the fitted EIS spectrum).

The development of immunosensors requires the integration of building blocks that are electrochemically stable and reproducible in the standard biocompatible media of confirmed physiological relevance. We carried out cyclic voltammetric (CV) and square-wave voltammetric (SWV) characterization of unmodified and APTES-modified Bi_2_Ru_2_O_7_ clusters in (i) 0.1 M KCl (pH = 7.0) and (ii) 1.0 mM [Fe(CN)_6_]^3-/4−^ in 0.1 M KCl deposited on the substrate SPCE. The electrochemical behavior of the deposited pyrochlore Bi_2_Ru_2_O_7_ clusters in a redox-free medium, i.e., in 0.1 M KCl, revealed a dual (electro)catalytic nature described by OER in the anodic region and ORR in the cathodic region (Figure S4). In addition, the ruthenium ions’ irreversible and discrete surface oxidation was observed in the anodic region, with a peak potential at ca. +0.6 V during the first CV potential scan. It was observed that although multivalent metallic centers are present in the structure of the (electro)catalytic material, no distinct internal redox system was present after the first CV scan. Notably, due to the absence of APTES, the adhesion of the pyrochlore clusters on the substrate electrode surface was somewhat poor, leading to their leaching. The leaching was visually noticeable already upon the addition of an electrolyte drop on the pyrochlore-modified SPCE surface before the measurement; thus, no further tests were carried out. The electrochemical behavior of APTES-Bi_2_Ru_2_O_7_ clusters in 0.1 M KCl was different compared to the nonmodified Bi_2_Ru_2_O_7_ clusters, with higher anodic currents for the OER reaction (Figure S4) and a signal for ORR that is separated from the hydrogen evolution reaction (HER) observed at more negative potentials. Importantly, the introduction of APTES resulted in a stable surface with no leaching, thus enabling its further investigation and modifications. Moreover, several advantageous characteristics were observed; i.e., the electrode modified with APTES-Bi_2_Ru_2_O_7_ clusters exhibited a diffusion-controlled behavior in 1.0 mM [Fe(CN)_6_]^3-/4−^ in 0.1 M KCl characterized by a linear relationship between the peak current signals and the square root of the scan rate, fast electron transfer kinetics, and high redox reversibility (Figure 4a,b). Upon the modification with APTES, the APTES-Bi_2_Ru_2_O_7_ clusters became firmly attached to the surface of the working SPCE, which was confirmed by running 30 consecutive CV (*i*_Pox_ = 47.8 ± 0.3 μA, *i*_Pred_ = −45.5 ± 0.3 μA) measurements in 1.0 mM [Fe(CN)_6_]^3−/4−^ in 0.1 M KCl (Figures 4c).

Notably, consecutive CV measurements showed that the leaching of the metallic ions from the APTES-Bi_2_Ru_2_O_7_ clusters and valence interplay between the metallic centers were not observed. In addition, the obtained EIS spectra revealed almost metallic conductivity of the SPCE modified with APTES-Bi_2_Ru_2_O_7_ clusters, with a small semicircle corresponding to the *R*_ct_ of ca. 180 Ω and a diffusion tail at lower frequencies (Figure 4d). The recorded spectra were fitted with a Randles-type equivalent circuit, shown in the inset of Figure 4d.

**Figure 5:**
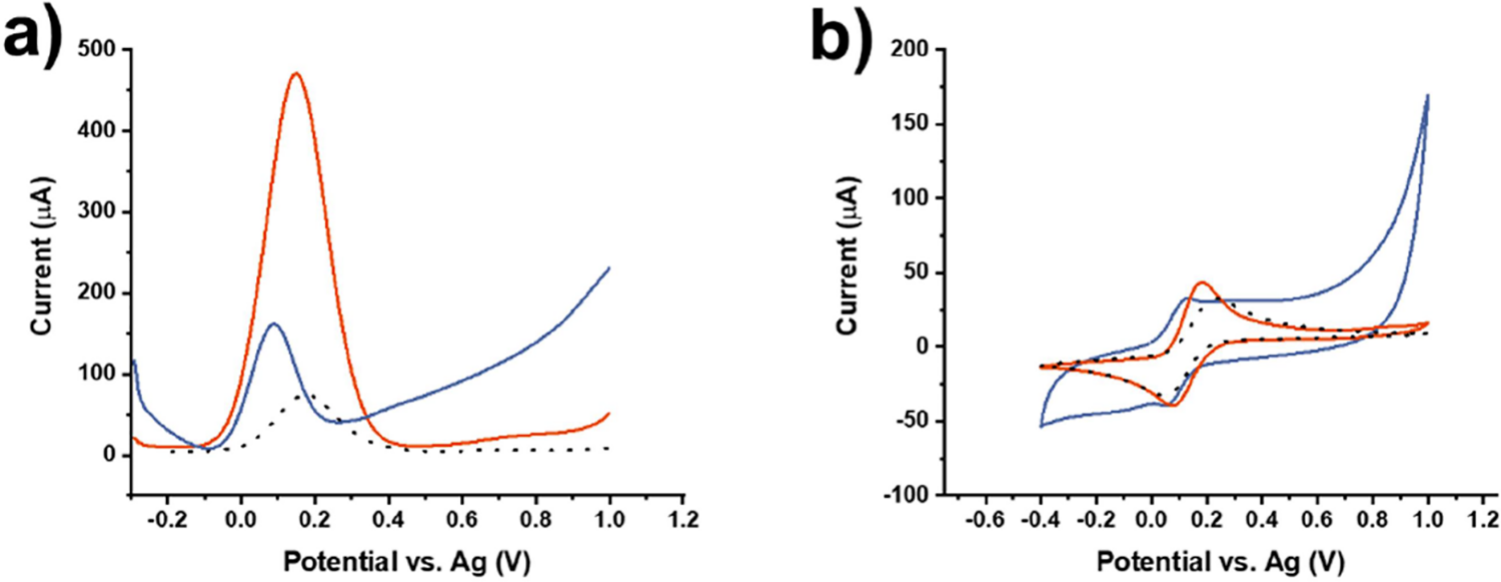
Superior behavior of APTES-Bi_2_Ru_2_O_7_ clusters. (a) SWV and (b) CV measurements in 1.0 mM [Fe(CN)_6_]^3−/4−^ in 0.1 M KCl carried out on unmodified SPCE (black-dashed), APTES (blue), and APTES-Bi_2_Ru_2_O_7_ cluster (red) modified SPCEs.

While the (electro)catalytic mechanism behind pyrochlore particles remains unsolved, it is hypothesized that pyrochlore reduces the kinetic barrier at active sites rather than engaging in electrochemical reactions with consumption, as demonstrated by Park et al.^7^ For example, the same study showed that pyrochlore outperforms IrO_2_ nanoparticles, despite the lower surface area. Indeed, in our study, the catalytic characteristics of Bi_2_Ru_2_O_7_ pyrochlore were observed when comparing bare SPCE, APTES-modified SPCE, and SPCE modified with APTES-Bi_2_Ru_2_O_7_ clusters (Figure 5). The SWV and CV measurements disclosed enhanced (electro)catalytic behavior of pyrochlore clusters reflected in a ca. 3-fold higher SWV response and well-defined reversible CV recording in 1.0 mM [Fe(CN)_6_]^3−/4−^ that was the external redox probe used also for all subsequent analytical performance tests.

Electrochemical studies proved favorable electrochemical and physical stability of the deposited APTES-Bi_2_Ru_2_O_7_ clusters without any observable valence interplay of the metallic centers (Figures 4, 5, and S4). From the stability studies in Figure 4c, it is evident that electrode pretreatment with HNO_3_ followed by APTES silanization of the Bi_2_Ru_2_O_7_ pyrochlore clusters leads to mechanically stable binding of the electrocatalytic material on the SPCE surface via −OH groups introduced by the pretreatment. Most importantly, the Bi_2_Ru_2_O_7_ clusters, in combination with APTES, yielded a substantial rise in the CV signal in the presence of an external redox probe, compared to the nonmodified electrodes and electrodes modified only with APTES (Figure 5). An explanation for this is that the oxygen vacancies facilitate the movement of electrons within the pyrochlore crystal lattice, similar to metal oxide semiconductors.^28^ The existence of oxygen vacancies alters the electronic structure of pyrochlore materials, likely resulting in the formation of mid-gap states, acting as charge carriers.

### Fabrication of the Immunosensor

**Figure 6:**
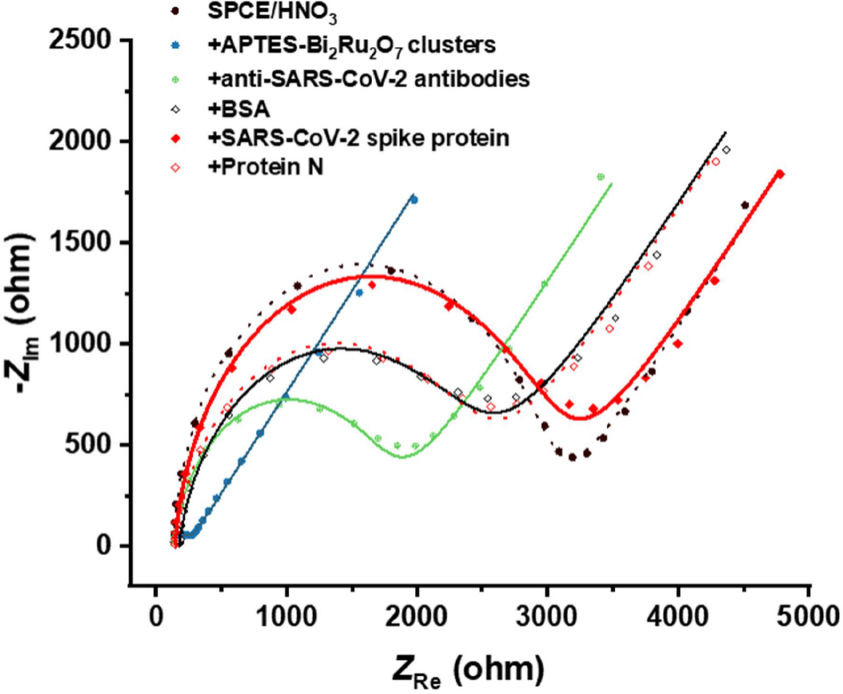
Step-by-step fabrication of the immunosensor. EIS (Nyquist) plots were recorded in 1.0 mM [Fe(CN)_6_]^3−^/^4−^ in 0.1 M KCl, along with the spike protein detection and negative control.

The deposition of APTES-Bi_2_Ru_2_O_7_ clusters on the SPCE substrate was followed by the immobilization of biorecognition elements. Efficient electrochemical immunosensing encompasses at least three key features, i.e., strong attachment of antibodies on the substrate electrode to minimize leaching, controlled site-specific binding to attain the desired orientation of the antibodies, and blockage of the nonspecific binding sites.^29^ Protein A possesses a high affinity for the Fc region of IgG immunoglobulins, enabling their efficient immobilization; more importantly, its use provides proper orientation of the antibodies.^30,31^ One protein A molecule has been shown to bind at least two molecules of IgG simultaneously.^32^ Such an approach enhances the sensitivity and selectivity of the immunosensor by assuring that the active binding sites on the antibodies’ Fab domains are readily accessible for the recognition event with the target spike protein. Notably, protein A can also serve as a blocking agent before using secondary antibodies in the case of immunohistochemistry to minimize nonspecific binding.^33^ This implies that the presence of protein A within the immunosensing architecture may also contribute by blocking nonspecific binding to a certain extent, thereby further enhancing the selectivity and improving the signal-to-noise ratio of the immunosensor. Before the attachment of protein A, we introduced GA as the cross-linker, exhibiting immediate positive effects, such as a higher degree of reproducibility of further fabrication steps and the generation of a strong analytical signal toward SARS-CoV-2 spike protein. Stepwise fabrication of the immunosensor was monitored by EIS (Figure 6) using 1.0 mM [Fe(CN)_6_]^3−/4−^ in 0.1 M KCl as a redox probe. The impedimetric behavior of bare SPCE is characterized by one semicircle related to *R*_ct_ and a typical diffusion tail.

The introduction of APTES-Bi_2_Ru_2_O_7_ clusters led to the system with the lowest *R*_ct_ and the prevailing diffusion pattern. After the immobilization of anti-SARS-CoV-2 antibodies via GA−protein A coupling, a higher *R*_ct_ value was observed. In the next step, the remaining nonspecific binding sites were blocked by adding BSA, and a further increase in *R*_ct_ characterized this surface modification. With this step, the fabrication of the immunosensor was completed. The other two signals shown in Figure 6 correspond to the negative control, i.e., incubation with protein N, showing a negligible difference versus the previous measurement, and a positive control, i.e., incubation with SARS-CoV-2 spike protein, demonstrating a significant increase in the *R*_ct_. The relative change between *R*_ct_ before and after incubation with the spike protein was used as the analytical signal.

**Figure 7:**
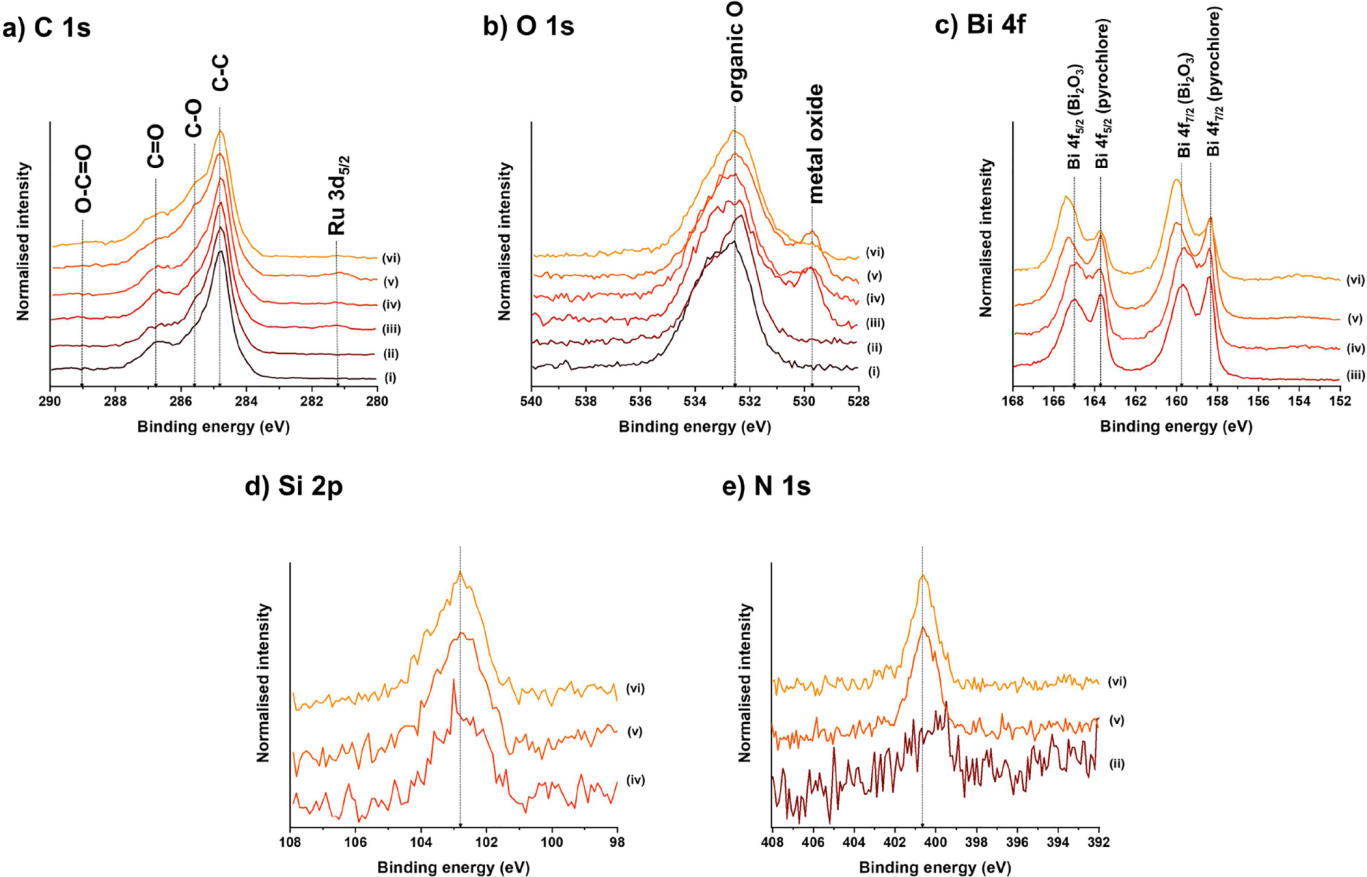
High-resolution XPS spectra for (a) C 1s and Ru 3d, (b) O 1s, (c) Bi 4f, (d) Si 2p, and (e) N 1s. The spectra were measured on different samples related to the gradual immunosensor fabrication: (i) bare SPCE, (ii) SPCE/HNO_3_, (iii) SPCE/HNO_3_/Bi_2_Ru_2_O_7_, (iv) SPCE/HNO_3_/APTES-Bi_2_Ru_2_O_7_, (v) SPCE/HNO_3_/APTES-Bi_2_Ru_2_O_7_/GA/protein A, and (vi) SPCE/HNO_3_/APTES-Bi_2_Ru_2_O_7_/GA/protein A/anti-SARS-CoV-2 antibodies.

More insights into the fabrication process were acquired by using the XPS technique, with a particular emphasis on protein stacking (Figure 7). XPS analysis confirmed the presence of C, Ru, Bi, N, O, and Si on the surface throughout the immunosensor preparation steps. The survey spectra for all steps reveal the surface composition of each sample and are shown in Figure S5. The C 1s spectra for all samples include C−C, C−O, C=O, and C=O, located at 284.8, 285.6, 286.6, and 289.0 eV, respectively (Figure 7a). These features are visible and well pronounced in all samples, with a notable increase in the C−O signal upon adding protein species, i.e., protein A and anti-SARS-CoV-2 antibodies. The addition of Bi_2_Ru_2_O_7_ clusters on the SPCE resulted in the formation of the Ru 3d_5/2_ signal located on the negative binding energy side of the C 1s spectra. Regarding the O 1s environment shown in Figure 7b (and Figure S6), several changes can be observed. Initially, the acidic treatment of SPCE resulted in a more pronounced C−OH feature at 532.3 eV. The addition of Bi_2_Ru_2_O_7_ clusters introduced a metal oxide feature at 529.8 eV, which gradually decreased with the subsequent addition of proteins covering the Bi_2_Ru_2_O_7_ moieties. Moreover, the Bi spectra shown in Figure 7c indicate two different environments of Bi, i.e., pyrochlore-related Bi 4f_5/2_ and Bi 4f_7/2_ features at 163.7 and 158.4 eV, respectively, along with Bi 4f_5/2_ and Bi 4f_7/2_ features at 165.0 and 159.7 eV, respectively, corresponding to Bi_2_O_3._^34−38^ The XPS analysis also revealed that after introducing proteins, the Bi 4f_5/2_ and Bi 4f_7/2_ peaks for pyrochlore decreased relative to Bi 4f_5/2_ and Bi 4f_7/2_ peaks for Bi_2_O_3_, suggesting that Bi_2_O_3_ had not been noticeably modified and that the pristine pyrochlore mostly accounts for further modification with APTES and subsequent protein stacking. The functionalization of clusters with APTES is evident in the spectra corresponding to the Si 2p feature (Figure 7d). As expected, no such peaks were observed for Bi_2_Ru_2_O_7_ clusters without APTES (Figure S5), whereas APTES-Bi_2_Ru_2_O_7_ clusters showed a strong feature at around 103.0 eV. This APTES-related signal remained present with the subsequent addition of protein A and anti-SARS-CoV-2 antibodies.

Finally, the N 1s environment, in both normalized (Figure 7e) and non-normalized (see Figure S6) spectra, is strongly associated with the protein content. The low-intensity peak observed in the sample for SPCE treated with nitric acid is attributed to the presence of NO_3_^−^ residues. On the other hand, the progressive increase in N 1s signals in the XPS spectra with the addition of protein A and antibodies (along with the concurrent decrease of the Bi 4f_5/2_ and Bi 4f_7/2_ signals for pyrochlore) further confirmed the stacking of proteins on the pyrochlore clusters.

**Figure 8:**
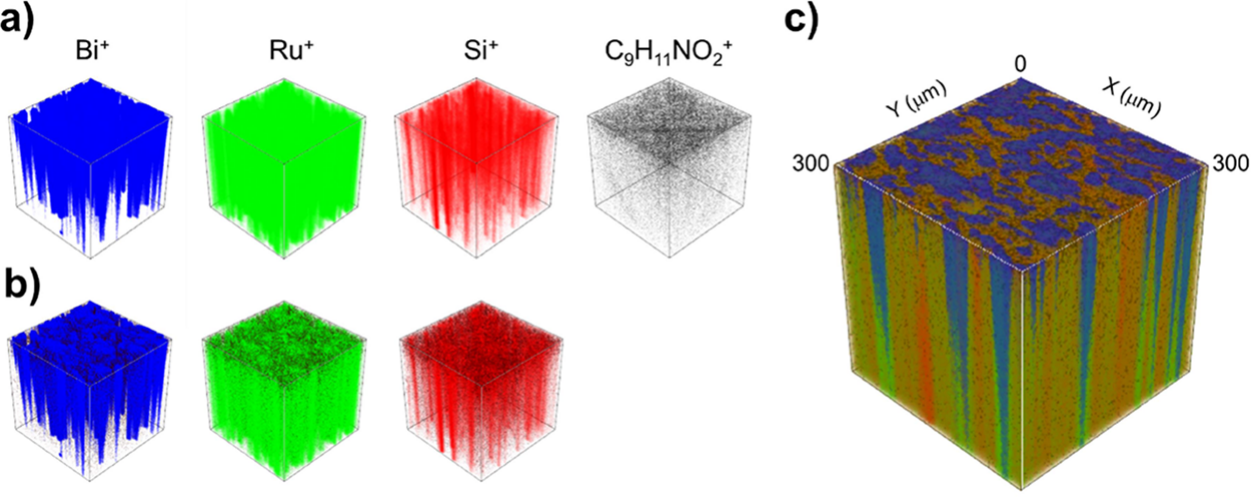
ToF-SIMS analysis of the immunosensing architecture. (a) 3D ToF-SIMS images showing the distribution of the main constituents, (b) an overlay of Bi^+^ (blue), Ru^+^ (green), and Si^+^ (red) signals with the signal for C_9_H_11_NO_2_^+^ (black), and (c) an overlay of all signals to represent the complete immunosensor architecture.

Along with the XPS, we performed a TOF-SIMS depth profiling analysis of the immunosensing architecture after the addition of anti-SARS-CoV-2 antibodies. The depth profiles of the signals characterizing different components for immunosensor construction are shown in Figure S7, while the 3D ToF-SIMS images showing the spatial distribution of components are presented in Figure 8. The immunosensor surface reveals a well-dispersed distribution of clusters, evidenced by both Ru^+^ and Bi^+^ signals, exhibiting an even surface coverage that is crucial for optimal functionality. The TOF-SIMS analysis further indicates that APTES closely follows the spatial distribution of these clusters, suggesting a uniform attachment across the surface. In addition, phenylalanine acts as a beacon for the proteins, i.e., the C_9_H_11_NO_2_^+^ moiety, facilitating their detection and interaction pattern. This amino acid is ubiquitously present on the immunosensor, as shown in Figure 8a, separately and with respect to Bi^+^, Ru^+^, and Si^+^ signals; the overlay ToF-SIMS 3D image is depicted in Figure 8b. Another identification of proteins on the topmost position (ToF-SIMS has an analyzed sampling depth of about 2 nm) comes from the presence of the signal for S^−^ corresponding to cysteine residues, which are confirmed through the analysis in negative ToF-SIMS polarity, as illustrated in Figure S8.

### Analytical Performance of the Impedimetric Immunosensor

**Figure 9:**
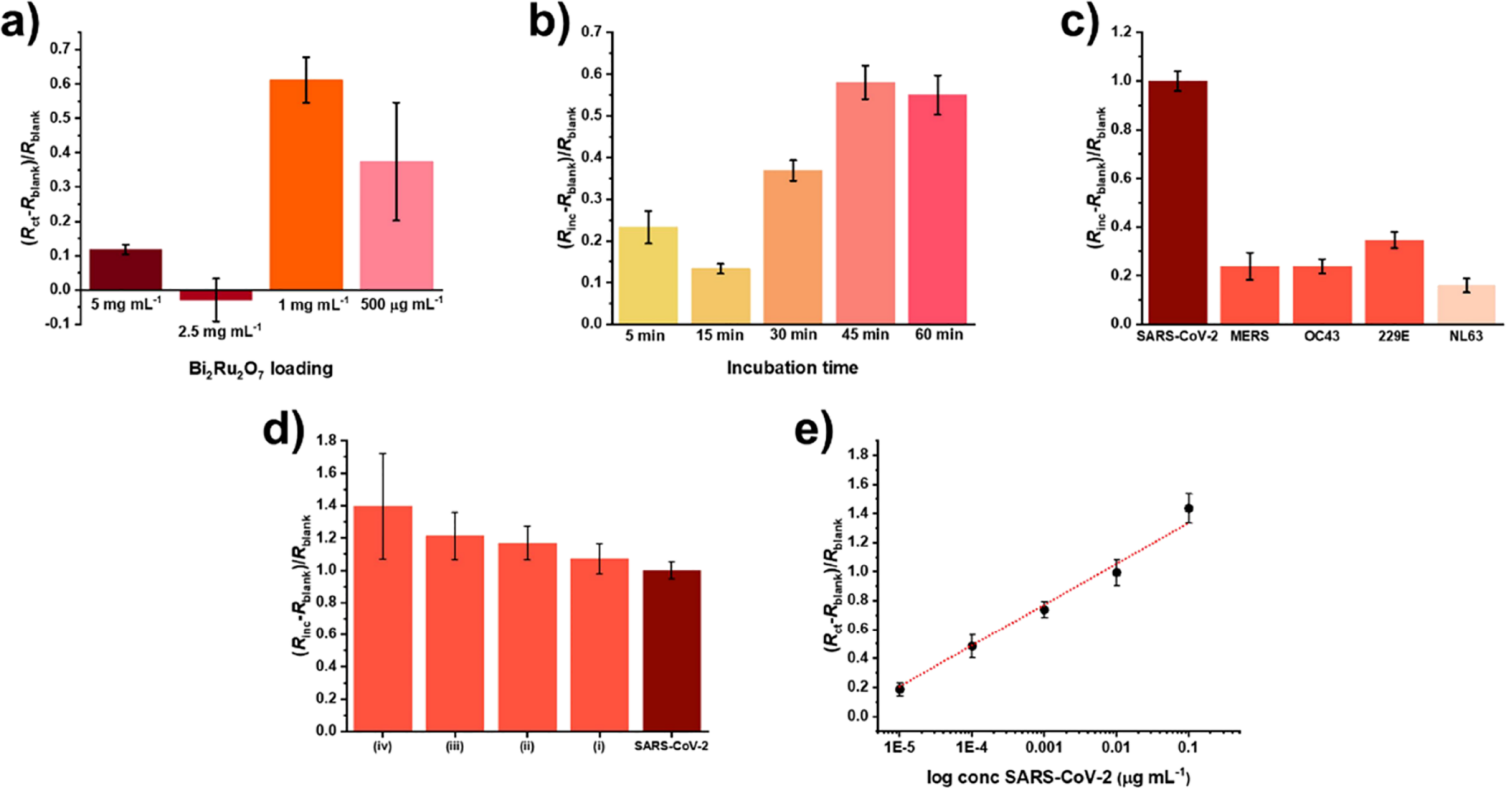
Optimization of the immunosensor and analytical performance. (a) Effect of the APTES-Bi_2_Ru_2_O_7_ cluster loading on the EIS signal for 1 ng mL^−1^ SARS-CoV-2 spike protein in PBS, (b) optimization of the SARS-CoV-2 spike protein incubation time in PBS (1 ng mL^−1^ SARS-CoV-2 spike protein in PBS), (c) signals obtained in the presence of spike proteins of various coronaviruses in ANF (30 min incubation, 1 mg mL^−1^ APTES-Bi_2_Ru_2_O_7_ loading, 1 ng mL^−1^ spike proteins), (d) signals obtained in the spike protein mixtures in ANF: (i) SARS-CoV-2 + MERS, (ii) SARS-CoV-2 + MERS + 229E, (iii) SARS-CoV-2 + MERS + 229E + NL63, (iv) SARS-CoV-2 + MERS + 229E + NL63 + OC43 (30 min incubation, 1 mg mL^−1^ of APTES-Bi_2_Ru_2_O_7_ loading, 1 ng mL^−1^ spike proteins), and (e) calibration obtained using optimal operational conditions in ANF samples; the corresponding EIS Nyquist spectra are shown in Figure S9. All measurements were carried out using 1.0 mM [Fe(CN)_6_]^3-/4−^ in 0.1 M KCl.

To study the effect of different Bi_2_Ru_2_O_7_ pyrochlore loadings on the SPCE surface, we applied different pyrochlore modification solutions, i.e., 0.5, 1.0, 2.5, and 5.0 mg mL^−1^, and followed the signal for 1.0 ng mL^−1^ of the SARS-CoV-2 spike protein. The signals significantly decreased at higher concentrations of 2.5 and 5.0 mg mL^−1^ pyrochlore, whereas the concentration of 1 mg mL^−1^ was selected as optimal (Figure 9a). The superior electroanalytical performances at 1.0 and 0.5 mg mL^−1^ of APTES-Bi_2_Ru_2_O_7_ clusters can be attributed to several factors. At these concentrations, the pyrochlore is well-dispersed over the supporting SPCE, maximizing the active surface area available for the interaction with the GA cross-linker and the functional proteins, as shown in Figure S9. This uniformity ensures efficient electron transfer and enhances the electrochemical response of the immunosensor. In contrast, at higher concentrations of 2.5 and 5.0 mg mL^−1^ APTES-Bi_2_Ru_2_O_7_ clusters, the (electro)catalytic material aggregates, leading to less uniform deposition. Such aggregation can result in a reduced effective surface area, hindering the electron transfer process and thus diminishing the analytical signal. In addition, we evaluated five different incubation times of the SARS-CoV-2 spike protein, i.e., 5, 15, 30, 45, and 60 min, to identify the duration yielding the highest response (Figure 9b). While a 5 min incubation time produced a slightly higher signal than a 15 min incubation, signal height significantly increased at 30 min incubation and at longer incubation times. Notably, the signal reached a maximum after 45 min incubation, with a minimal decrease observed after 60 min incubation. The incubation time study suggests that the binding interaction between the target protein and the immunosensor improves with a longer incubation time up to a certain point. The maximum signal observed after 45 min incubation indicates that the binding sites of the immunosensor surface have likely become saturated with the analyte protein. However, from a practical perspective, the 30 min incubation time represents an optimal balance between attaining a satisfactorily high electroanalytical signal and maintaining a reasonable assay duration. Shorter incubation times, such as 5 or 15 min, may not allow sufficient protein binding, leading to suboptimal immunosensing performance. To assess the selectivity of the optimized immunosensor toward SARS-CoV-2, we conducted measurements using spike proteins from various coronaviruses and their combinations, including MERS-CoV, HCoV-229E, HCoV-NL63, and HCoV-OC43 (Figures 9c,d).

The results proved that the immunosensor exhibited good selectivity for SARS-CoV-2, as evidenced by the considerably highest electroanalytical signal compared with the other coronaviruses, although a certain degree of interference could be observed for all tested proteins. Such performances can be attributed to an effective match between the selected antibody−antigen pair, alongside the efficient immobilization of the antibodies and blocking of the nonspecific binding sites. The relatively low interference observed for the selected HCoV spike proteins can be attributed to the similarity of structural motifs, such as the HCoV-229E spike protein, which may cause cross-reactivity with anti-SARS-CoV-2 antibodies. Although the RBD domains of all coronaviruses are different, similarities in other regions of the S1 subunit could result in a partial binding affinity. All measurements were performed in triplicate against the 1 ng mL^−1^ SARS-CoV-2 spike protein in ANF.

Finally, the immunosensor’s performance was tested across a wide concentration range of 10^−5^−10^−1^ μg mL^−1^ (corresponding to 130 fM to 1.3 nM) SARS-CoV-2 spike protein in ANF, as evidenced by the resulting linear calibration curve with a correlation coefficient (*r*^2^) of 0.98 (Figure 9e). Each calibration point, including blanks, was measured in four replicates using four identically prepared immunosensors per measuring point.

Apart from a wide operational concentration range, the optimized immunosensor exhibited a low detection limit (3σ criterion) of only 118 fM obtained in ANF with a relatively complex matrix. These results are competitive or surpass the performances of many reported impedimetric biosensors for the detection of the spike protein, including gold electrode incorporating a specific peptide capture probe with the detection limit of 1.28 nM,^39^ multiwalled carbon nanotubes functionalized with methylene blue on SPCE with the detection limit of 0.255 nM,^40^ ACE2 or CD147 functionalized screen-printed gold electrodes with the detection limit of 0.498 nM,^41^ electrodeposited gold nanoparticles functionalized with mercaptoacetic acid monolayers on the supporting SPCE with the detection limit of 3.16 pM,^42^ V_2_CT_*x*_ MXene-based immunosensors developed by our group with the detection limit of 45 fM in PBS,^43^ screen-printed gold electrode incorporating the chicken IgY antispike antibody with the detection limit of 72.2 fM in PBS,^44^ or SPCE modified with the metal−organic framework and silica with the detection limit of 1.27 fM in a nasal sample.^45^

## Conclusions

In this work, we demonstrate the synergistic effects of the (electro)catalytic nature of pyrochlore, the suitable stabilization and immobilization of pyrochlore by APTES, and the ability of APTES-modified Bi_2_Ru_2_O_7_ clusters as efficient domains for stacking functional proteins to develop a highly sensitive and selective immunosensor. CV and SWV measurements revealed improved electrochemical and physical stability of the APTES-Bi_2_Ru_2_O_7_ clusters without any observable valence interplay between the Ru and Bi centers. Presumably, this electrochemical activity is due to oxygen deficiency and metallic-like conductivity, which contributes to the increase of electron pathways for the external redox reaction, the introduction of additional active sites, and facilitating electron transfer. After stacking protein A and antibodies, the immunosensor exhibited high selectivity, owing to effective matching between the selected antibody−antigen pair, along with the efficient immobilization of the antibodies and blocking of the nonspecific binding sites. The high sensitivity and overall electroanalytical performance, characterized by high linearity in the investigated concentration range and a detection limit of 118 fM SARS-CoV-2 spike protein, underline the advantageous application of pyrochlores in the development of high-performance immunosensors.

## Materials and Methods

### Synthesis and Characterization of Bi_2_Ru_2_O_7_

A stoichiometric mixture of α-Bi_2_O_3_ (Fluka, 99.8%) and RuO_2_ × H_2_O (Acros Organics, >54% Ru) was dry homogenized for about 30 min in an agate mortar, heated at 900 °C for 3 h (heating rate 4 °C min^−1^) in an open Pt-crucible in a chamber furnace, and then cooled down to room temperature. The obtained powder was examined on an Ital Structure APD 2000 X-ray powder diffractometer using Cu *K*α radiation (λ = 1.5418 Å) in the 2θ range from 20 to 80° with a step width of 0.02° and a counting time of 1 s per step.

FE-SEM images and EDX analysis of pyrochlore clusters deposited on the SPCE surface were obtained with a high-resolution scanning electron microscope (Carl Zeiss SUPRA 35 VP FE-SEM) equipped with an energy-dispersive X-ray spectrometer (Oxford Instruments Inca 400 EDX). Bare and SPCEs modified with APTES-Bi_2_Ru_2_O_7_ clusters were subjected to optical inspection using 3D profilometry (Zegage PRO HR, Zygo Corporation, PA).

X-ray photoelectron spectroscopy (XPS) analyses were conducted using a Supra+ system from Kratos (Manchester, UK) with an Al K_α_ source as the excitation source. The spectra were referenced by aligning the C−C/C-H peak in the C 1s to 284.8 eV. The SPCEs were mounted on the sample holder by using double-sided carbon tape. Measurements were conducted at a takeoff angle of 90°, focusing on a spot size of 300 × 700 μm, with the pass energy set at 20 eV. ESCApe 1.5 software from Kratos was utilized for data collection and analysis.

For the ToF-SIMS experiments, an M6 instrument from Iontof (Münster, Germany) was utilized to perform the measurements. The primary ion beam used was a Bi^+^ beam with an energy of 30 keV and the target current set at 1.0 pA. Surface Lab 7.3 software, provided by Iontof, was employed for both data acquisition and analysis. Calibration of the spectra was achieved by referencing known ion signals at specific *m*/*z* values. To address any potential charging issues during the measurements, a flood gun was turned on. Silicon-free double-sided tape was used to secure SPCEs onto a top-mounted sample holder. Depth profiling was carried out using 250 kV Cs^+^ ions, operating at a target current of 10 nA. The sputter area was 500 by 500 μm, and the analysis was performed within the center of the sputter crater, targeting a spot size of 300 by 300 μm.

### Immunosensor Fabrication

The working electrode of the SPCE (Metrohm, DRP-110, *d* = 4 mm, shown in Figures S1 and S10) was treated with 0.5 M HNO_3_ for 15 min to activate its surface by introducing −OH groups. Afterward, the electrode was rinsed with ultrapure H_2_O and dried with nitrogen under a mild gas flow. Then, 10 μL of APTES-modified Bi_2_Ru_2_O_7_ powder was applied to the working electrode by drop-casting and left at 35 °C for 1 h to dry completely. Previously, the APTES-Bi_2_Ru_2_O_7_ clusters were prepared using the following procedure: the synthesized Bi_2_Ru_2_O_7_ was dispersed in absolute ethanol to form a 5 mg mL^−1^ suspension followed by adding 5 vol % of APTES. After 2 h of vigorous stirring at room temperature, the mixture was centrifuged twice for 3 min at 11000 rpm while rinsing with absolute ethanol. After the second centrifugation, followed by rinsing, the activated powder was resuspended in distilled H_2_O to make a 5 mg mL^−1^ solution. The working electrode modified with APTES-Bi_2_Ru_2_O_7_ clusters was covered with 10 μL of PBS (pH 7.4) containing 5% GA, and the electrode was left at 4 °C for 2 h to promote the formation of the GA cross-link. Then, the electrode was rinsed by immersion into distilled H_2_O for 10 s and dried under a mild nitrogen gas flow. This was followed by covering the electrode with 10 μL of 5 μg mL^−1^ protein A in PBS (pH 7.4) and left for 1 h at room temperature (22−25 °C) under dark storage conditions. The electrode was then rinsed with distilled H_2_O, dried with nitrogen, and incubated with 10 μL of 10 μg mL^−1^ anti-SARS-CoV-2 antibodies in PBS (pH 7.4) for 1 h at room temperature. To minimize the nonspecific binding, the remaining cross-links and other nonspecific binding sites were blocked by adding 10 μL of 100 μg mL^−1^ BSA solution in PBS (pH 7.4) on the working electrode and left at room temperature for 1 h. After a short rinsing sequence with Tris buffer, the immunosensor was dried with nitrogen and was ready for use. The complete fabrication procedure is depicted in Scheme S1.

### Electrochemical Measurements

All measurements were carried out using a portable PalmSens 4 potentiostat/galvanostat operated by PSTrace 5.9 (PalmSens BV, The Netherlands). Electrochemical impedance spectroscopy (EIS) was carried out in a frequency range of 10^4^−10^−1^ Hz at the potential of +0.14 V vs a screen-printed quasi-reference silver electrode and an amplitude of 5 mV in solution containing 1.0 mM [Fe(CN)_6_]^3−^/^4−^ in 0.1 M KCl as a redox probe.

The recorded EIS spectra were fitted using the equivalent electrical circuit *R*_S_([*R*_ct_*Z*_W_]*Q*_dl_). Here, *R*_S_ corresponds to the solution resistance, *R*_ct_ is the charge transfer resistance of the outer sensing layer, *Z*_W_ is a Warburg element that models the diffusion phenomena, and *Q*_dl_ is a constant phase element used to model nonideal double-layer capacitance.

## Supporting Information Available

Chemicals, preparation of buffer solutions, fabrication of the immunosensor; Scheme S1, immunosensor fabrication protocol; Figure S1, 3D interferometry image of SPCE; Figure S2, FE-SEM image of the APTES-Bi_2_Ru_2_O_7_ cluster; Figure S3, EDX analysis of APTES-Bi_2_Ru_2_O_7_ clusters; Figure S4, cyclic voltammograms of modified SPCEs; Figure S5, XPS survey spectra; Figure S6, non-normalized XPS spectra for N 1s and O 1s; Figure S7, depth profiles measured in positive-ion TOF-SIMS polarity; Figure S8, detailed negative-ion ToF-SIMS spectrum showing the S^−^ signal; Figure S9, EIS Nyquist spectra for different concentrations of the SARS-CoV-2 spike protein in ANF; and Figure S10, 3D and 2D interferometry images of bare and APTES-Bi_2_Ru_2_O_7_-modified SPCEs (PDF)
