# Supplementary material for: Protein Stacking on the APTES-Functionalized Pyrochlore Bi2Ru2O7 Clusters for Ultrasensitive and Selective Immunosensing
Source: ACS Appl Mater Interfaces. 2025 Feb 6;17(7):10792–801. doi: 10.1021/acsami.4c17869 (PMC11843530; doi:10.1021/acsami.4c17869)
Supplement: Supplementary file 1 — am4c17869_si_001.pdf [file am4c17869_si_001.pdf]

Supporting Information

Protein Stacking on the APTES-Functionalized Pyrochlore $Bi_2Ru_2O_7$ Clusters for Ultrasensitive and Selective Immunosensing

Nikola Tasić[1,*], Nika Vranešič[1], Dino Metarapi[1], Kristina Mervič[1], Milan Žunić[2], Aleksandra Dapčević[3], Matjaž Finšgar[4], Samo B. Hočevar[1,*]

[1]*Department of Analytical Chemistry, National Institute of Chemistry, Hajdrihova 19, 1000 Ljubljana, Slovenia*

[2]*University of Belgrade, Institute for Multidisciplinary Research, Kneza Višeslava 1, 11030 Belgrade, Serbia*

[3]*Department of General and Inorganic Chemistry, Faculty of Technology and Metallurgy, University of Belgrade, Karnegijeva 4, 11000 Belgrade, Serbia*

[4]*Faculty of Chemistry and Chemical Engineering, University of Maribor, Smetanova 17, 2000 Maribor, Slovenia*

[*]nikola.tasic@ki.si

[*]samo.hocevar@ki.si

Chemicals

All commercial reagents were of analytical grade and were used without further purification. All aqueous solutions were prepared using ultrapure water with resistivity not less than 18.2 MΩ cm at 298 K (Milli-Q, Millipore, Corp., Marlborough, USA).

Potassium hexacyanoferrate(III) ($K_3Fe(CN)_6$), glutaraldehyde solution (25% in water), Tris base (tris(hydroxymethyl)aminomethane, $C_4H_{11}NO_3$), and APTES (3-aminopropyl)triethoxysilane) were purchased from Sigma Aldrich, sodium azide (NaN_3) and bovine serum albumin (BSA) were purchased from Fluka, potassium hexacyanoferrate(II) trihydrate ($K_4Fe(CN)_6{\cdot}3H_2O$) was purchased from Riedel de Haen, potassium chloride (KCl), sodium chloride (NaCl), sodium hydrogen phosphate dihydrate ($Na_2HPO_4{\cdot}2H_2O$), and potassium dihydrogen phosphate (KH_2PO_4) were purchased from Merck, absolute ethanol (C_2H_5OH) was purchased from Carlo Erba. Human monoclonal anti-SARS-CoV-2 spike glycoprotein antibodies (ab286179) were purchased from Abcam, and spike protein from Bio Bench. HCoV-OC43 spike protein (40607-V08H1), HCoV-HKU1 spike protein (40021-V08H), HCoV-NL63 spike protein (40600-V08H), HCoV-229E spike protein (40605-V08B), and MERS spike protein (40069-V08B) were all purchased from Sino Biological Europe GmbH, Eschborn, Germany. The artificial saliva was purchased from BioChemazone.

Preparation of buffer solutions

Phosphate-buffered saline (PBS, pH=7.4) was prepared by dissolving 8.0 g NaCl, 200.0 mg KCl, 1.81 g $Na_2HPO_4{\cdot}2H_2O$, and 240.0 mg KH_2PO_4 in 1.0 L of purified H_2O. When the PBS was used to dilute proteins, 0.001% NaN_3 was added to the solution as a preservative. Tris buffer solution was prepared by dissolving 1.211 g Tris base (tris(hydroxymethyl)aminomethane; $C_4H_{11}NO_3$) in 500 mL of purified water in a flask with a magnetic stirrer for 30 min (720 rpm). The solution was stored at room temperature.

Fabrication of the immunosensor

Prior to all modifications, the supporting screen-printed carbon electrodes (SPCEs, DropSens, DRP-C110, and C-110, Metrohm, Herisau, Switzerland) were tested using square-wave voltammetry (SWV) in 1.0 mM $[Fe(CN)_6]^{3-/4-}$ in 0.1 M KCl. The electrodes were categorized based on their peak current response, and only those electrodes with similar response, i.e., relative standard deviation <10%, were used for further modifications and studies. The intrinsic irreproducibility of SPCEs originates from the fabrication procedure, which typically involves several steps and processing variables, such as the composition of the screen-printed paste and calcination profile, that contribute to the well-like structure and somewhat inhomogeneous electrode surface in the final product (see **Figure S1**). Each step of the immunosensor preparation was conducted with a different SPCE that was previously characterized to provide consistent electrochemical conditions during the study.

Scheme S1. Fabrication protocol for impedimetric SARS-CoV-2 spike protein immunosensor.

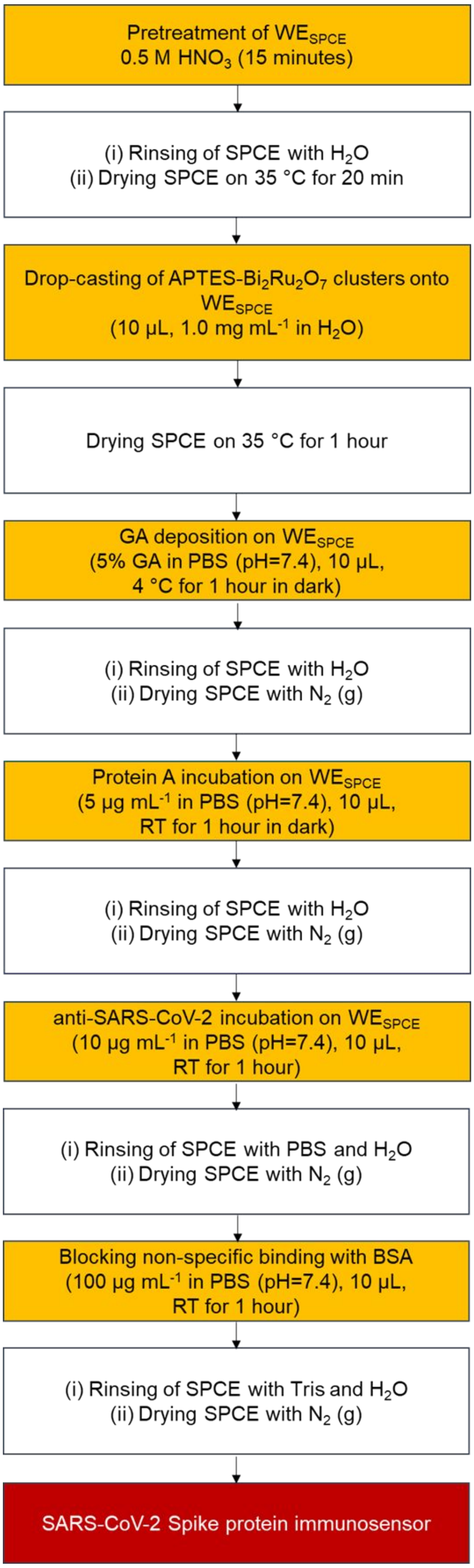

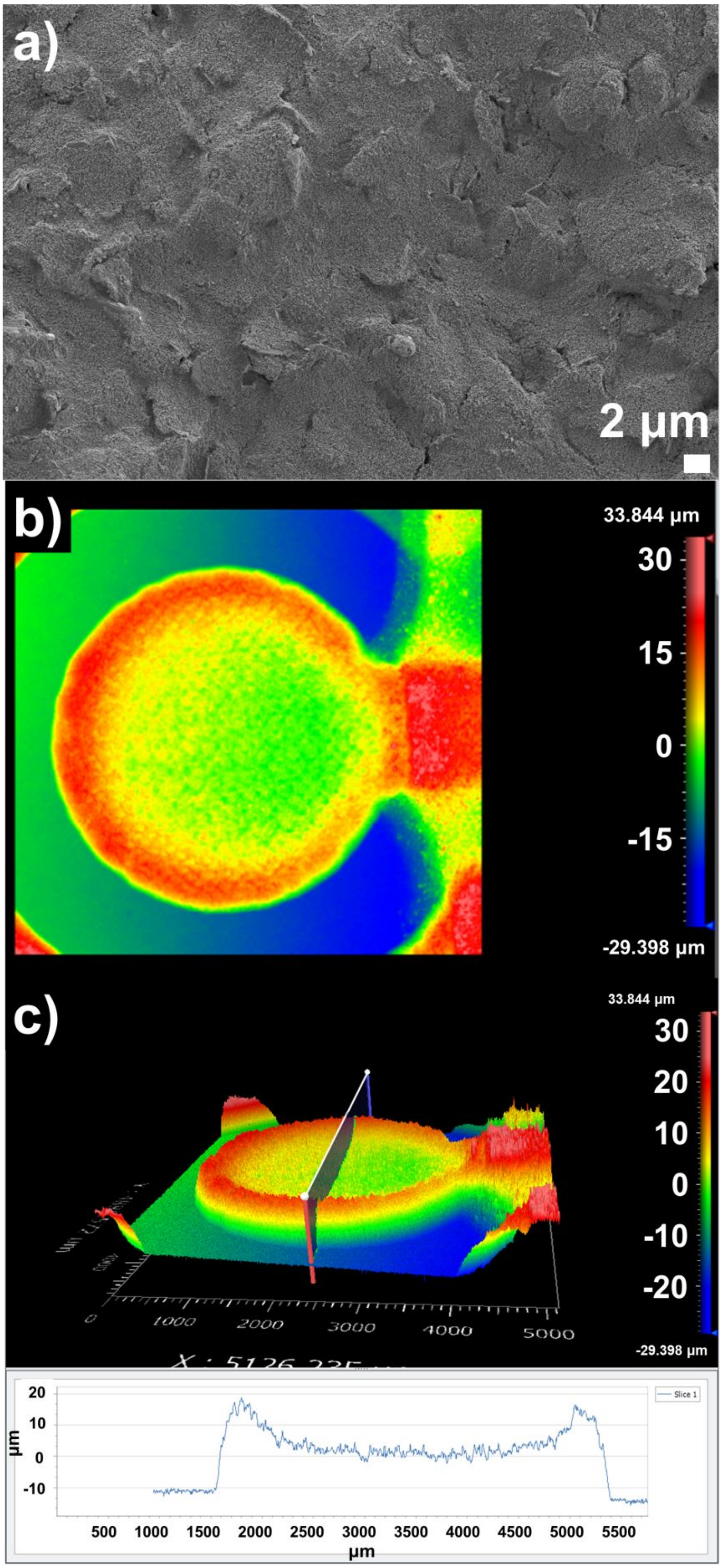

Figure S1. Surface heterogeneity of a well-like structured working electrode within the SPCE unit. (a) FE-SEM image of the SPCE surface, (b) top-view profilometry image of the SPCE disclosing a well-like structure of the working electrode surface, and (c) 3D profilometry image of the SPCE along with an arbitrary section profile.

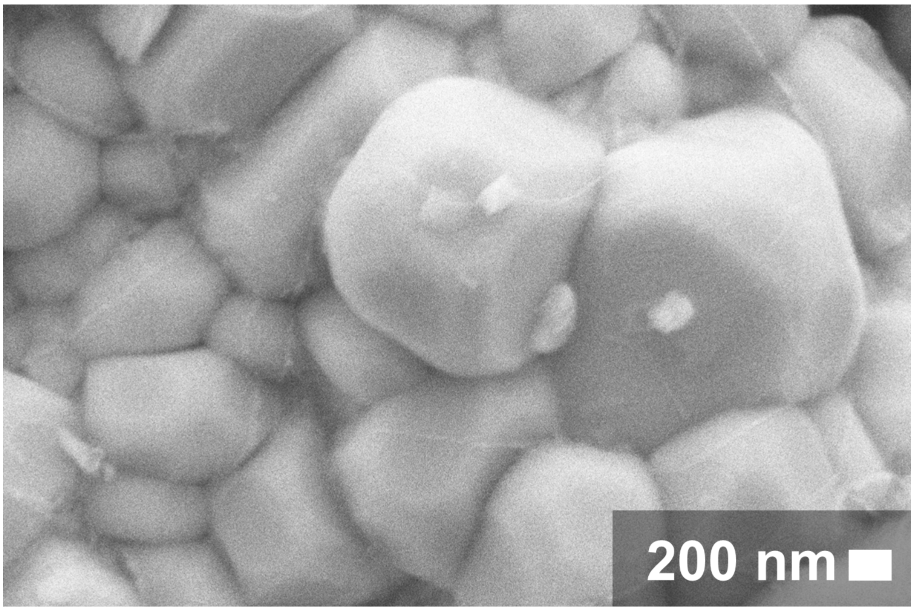

Figure S2. High-magnification FE-SEM image of APTES-$Bi_2Ru_2O_7$ cluster.

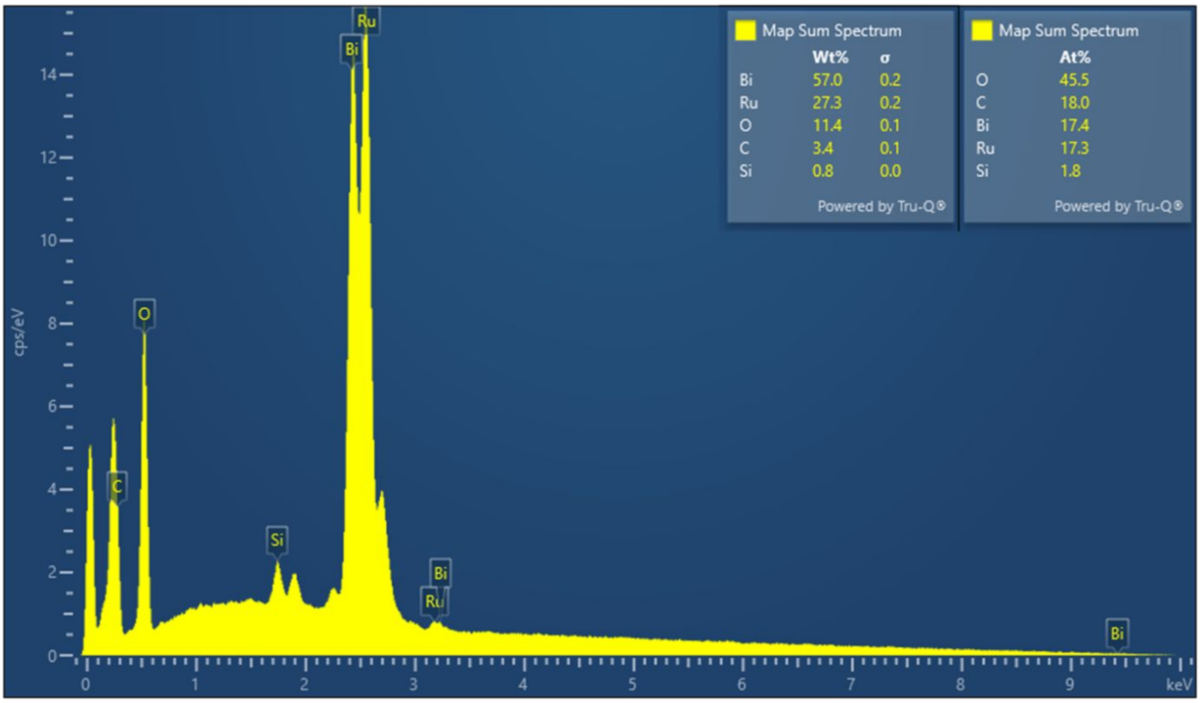

Figure S3. EDX analysis. Elemental composition (wt.%) of the APTES-$Bi_2Ru_2O_7$ clusters. The corresponding acquisition micrograph with elemental spatial distribution is presented in **Figure 3** in the manuscript.

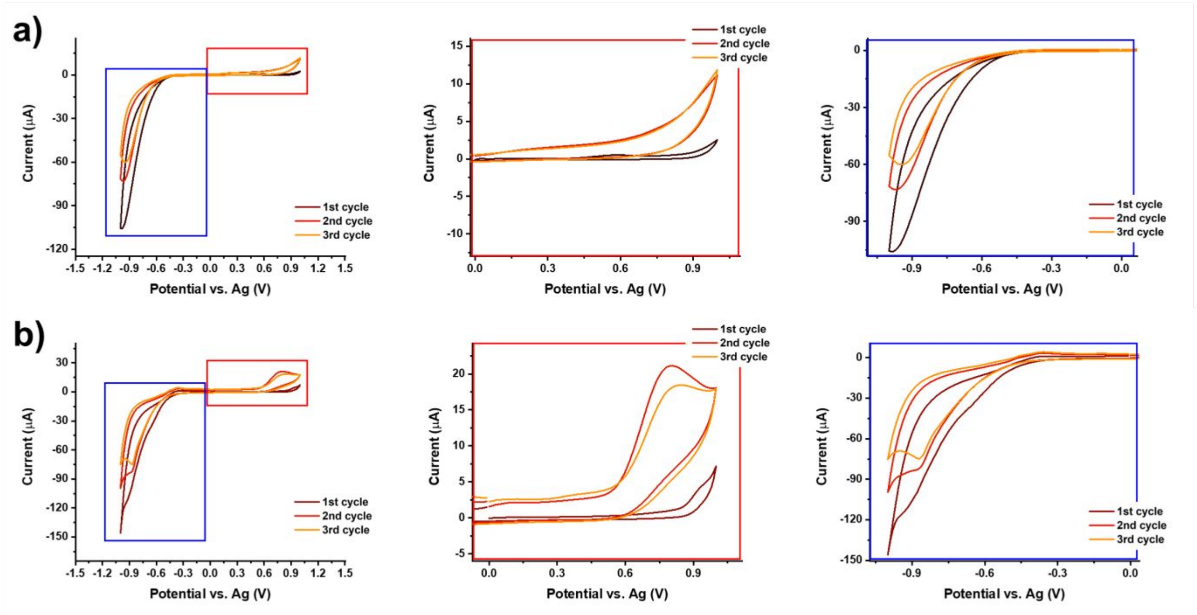

Figure S4. Cyclic voltammograms of SPCE modified with (a) $Bi_2Ru_2O_7$ and (b) APTES-$Bi_2Ru_2O_7$ clusters in 0.1 M KCl. The graphs show the entire investigated potential window, and magnified anodic and cathodic regions, respectively.

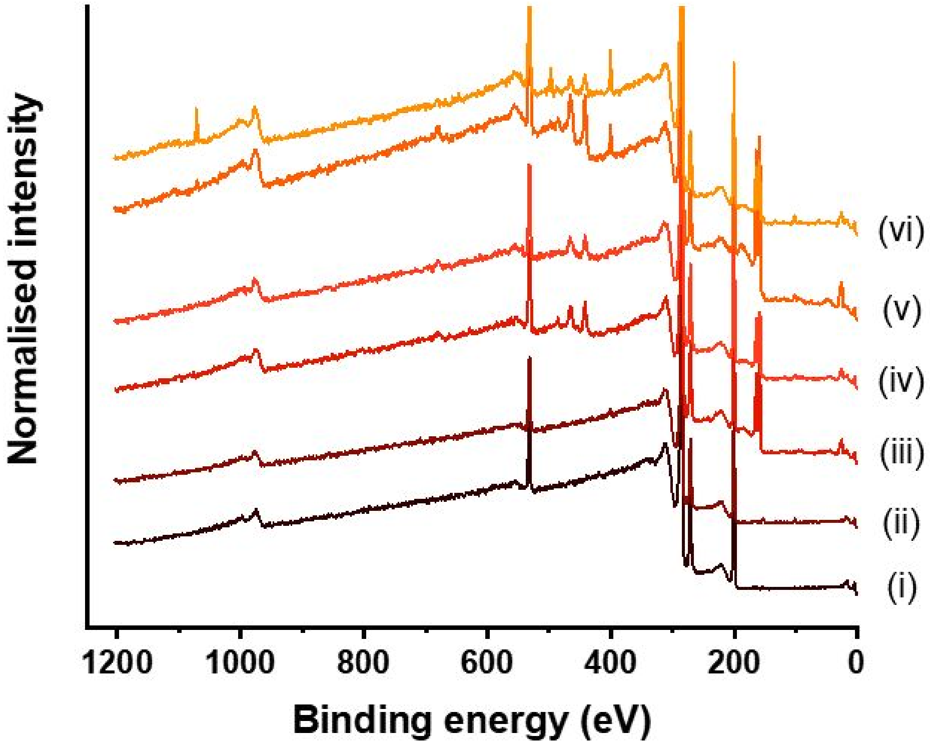

Figure S5. XPS survey spectra. Survey spectra measured for different samples (i) bare SPCE, (ii) SPCE/HNO_3, (iii) SPCE/HNO_3/$Bi_2Ru_2O_7$, (iv) SPCE/HNO_3/APTES-$Bi_2Ru_2O_7$, (v) SPCE/HNO_3/APTES-$Bi_2Ru_2O_7$/GA/Protein A, (vi) SPCE/HNO_3/APTES-$Bi_2Ru_2O_7$/GA/Protein A/anti-SARS-CoV-2 antibodies.

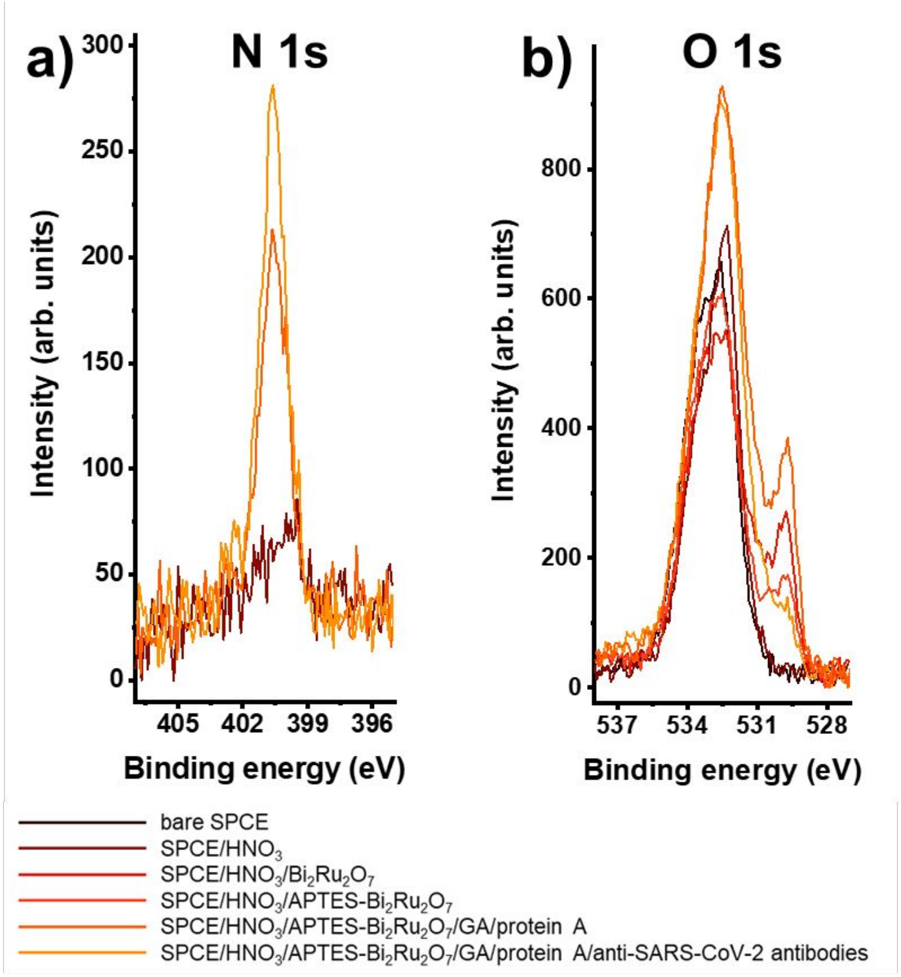

Figure S6. Non-normalized XPS spectra for (a) N 1s and (b) O 1s, in different samples.

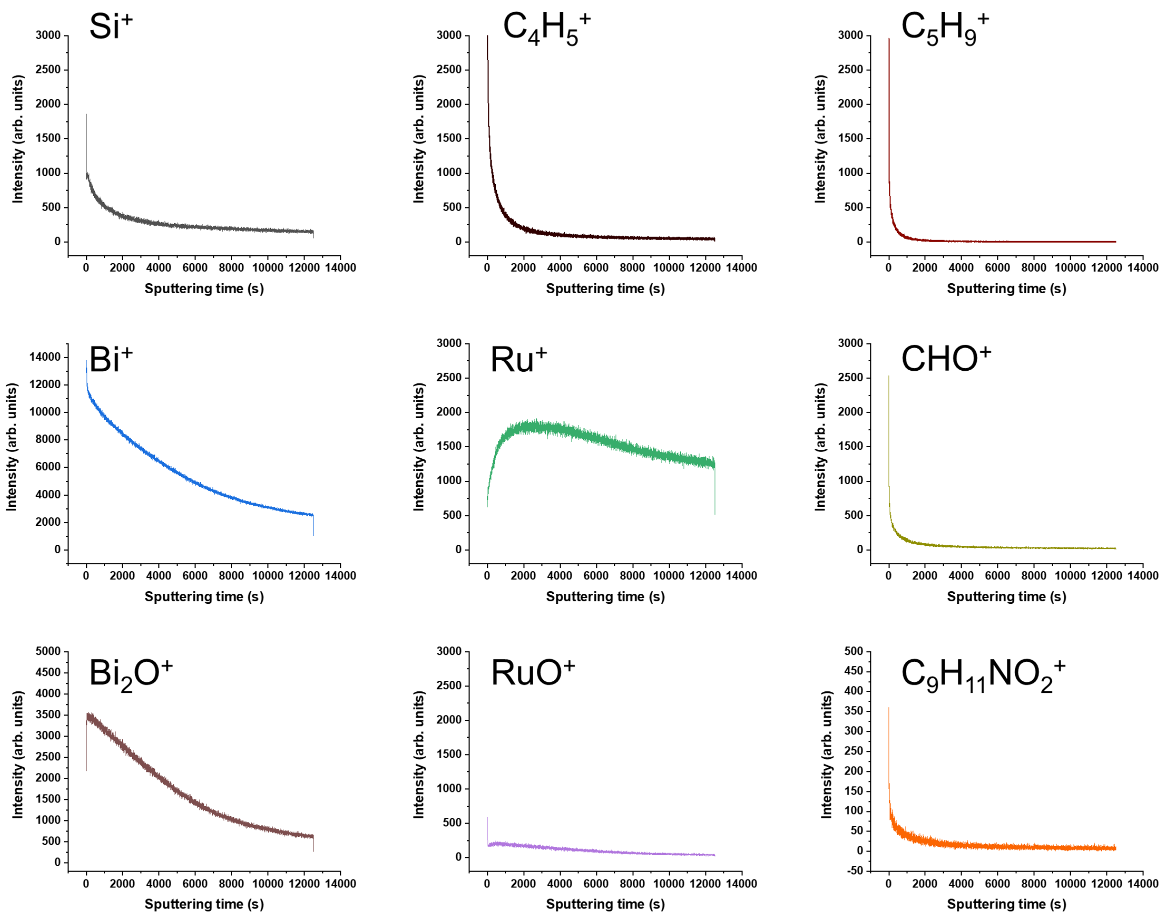

Figure S7. Depth profiles measured in positive-ion TOF-SIMS polarity.

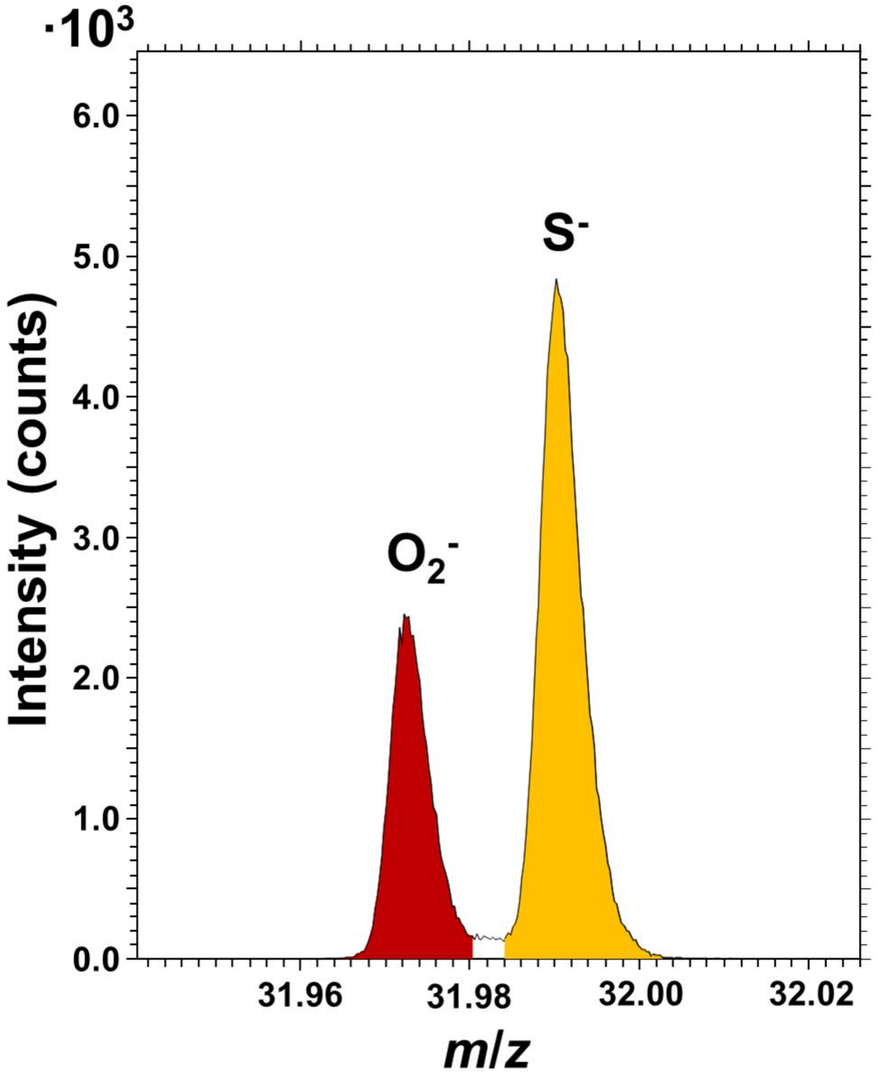

Figure S8. A detail in negative-ion ToF-SIMS spectrum showing the presence of S^- signal. This signal serves as a beacon for cysteine amino acid residues belonging to the stacked protein fragments.

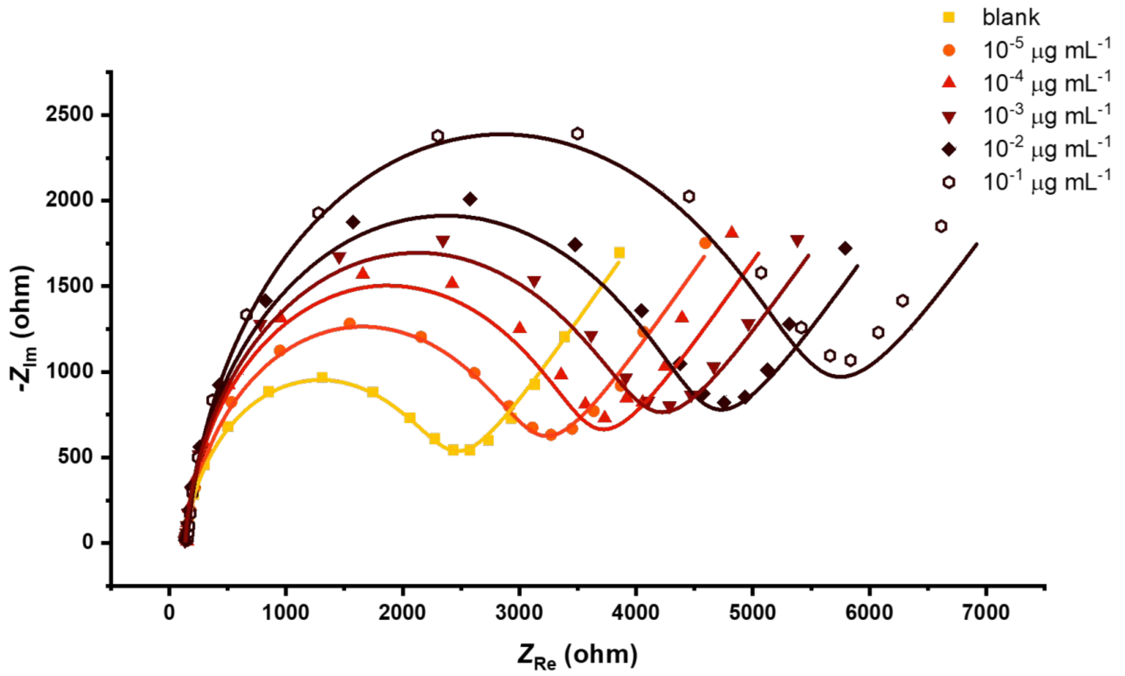

Figure S9. EIS Nyquist spectra recorded for different concentrations of SARS-CoV-2 spike protein in ANF. All measurements were carried out using 1.0 mM $[Fe(CN)_6]^{3-/4-}$ in 0.1 M KCl.

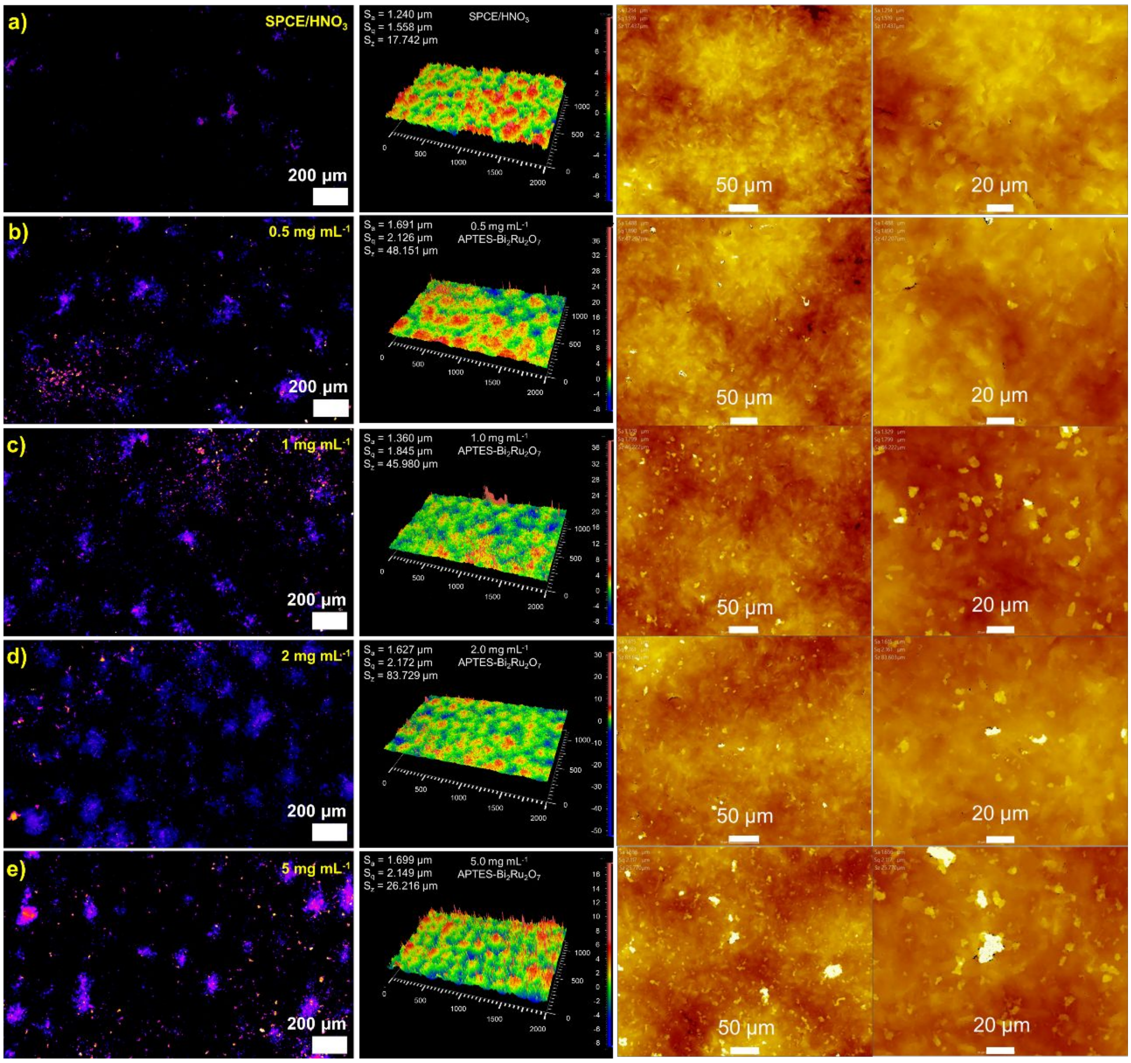

Figure S10. 3D and 2D interferometry images of (a) bare SPCE treated with HNO_3 and SPCEs modified with varying concentrations of APTES-$Bi_2Ru_2O_7$ in the drop-casting solution: (b) 0.5, (c) 1.0, (d) 2.0, and (e) 5 mg mL^{-1}. The images reveal significant surface heterogeneity of the supporting SPCEs. The 3D images highlight surface morphology changes induced by increasing concentration of the pyrochlore clusters on the electrode surface. Lower concentrations of 0.5 and 1.0 mg mL^{-1} exhibit better dispersion with smaller aggregates, whereas higher concentrations result in larger aggregates and less uniform dispersion, as seen in 2D high magnification images on the right.
